# Investigation of the relationship between ischemic stroke and endothelial nitric oxide synthase gene polymorphisms [G894T, intron 4 VNTR and T786C]

**DOI:** 10.3906/sag-1808-57

**Published:** 2019-04-18

**Authors:** Süleyman Ömer ANLIAÇIK, Serhat TOKGÖZ, Ayşe Gül ZAMANİ, Mahmut Selman YILDIRIM, Mehmet Sinan İYİSOY

**Affiliations:** 1 Department of Neurology, Meram Medical Faculty, Necmettin Erbakan University, Konya Turkey; 2 Department of Molecular Biology and Genetics, Meram Medical Faculty, Necmettin Erbakan University, Konya Turkey; 3 Department of Medical Education and Informatics, Meram Medical Faculty, Necmettin Erbakan University, Konya Turkey

**Keywords:** Stroke, polymorphism, endothelial nitric oxide synthase, G894T, variable number tandem repeat, T786C

## Abstract

**Background/aim:**

We aimed to investigate the associations between endothelial nitric oxide synthase (eNOS) gene polymorphisms [G894T (rs1799983)], intron 4 (27-bpTR) variable number tandem repeat (VNTR) and T786C (rs2070744), and ischemic stroke in the Anatolian population.

**Materials and methods:**

This case-control study included 112 patients with “stroke of undetermined etiology” and 160 controls. Real-time polymerase chain reaction (RT-PCR) analysis was used to analyze these polymorphisms. Between-group frequencies of alleles and genotypes were compared using binary logistic regression analysis.

**Results:**

No significant difference was observed between the two groups in terms of the genotype and allele distributions of the eNOS G894T (rs1799983) polymorphism (P > 0.05). The a alleles and the 4b/a and 4a/a genotypes of the intron 4 (27-bpTR) VNTR polymorphism had significantly higher frequencies in the patient group than in the control group (OR: 2.715, P < 0.001; OR: 3.396, P < 0.001; OR: 10.631, P = 0.016, respectively). On the contrary, the TC genotype and C alleles of the T786C (rs2070744) polymorphism had a significantly lower frequency in the patient group than in the control group (OR: 0.244, P < 0.001, OR: 0.605, P = 0.006, respectively).

**Conclusion:**

Our findings indicate that the eNOS G894T and T786C [rs2070744] polymorphisms are not associated with the risk of ischemic stroke, whereas the intron 4 [27-bpTR] VNTR may be a risk factor in the Anatolian population.

## 1. Introduction

According to the WHO, stroke is a clinical syndrome caused by vascular lesion. It is characterized by rapidly occurring clinical symptoms and/or signs of focal or generalized cerebral dysfunction lasting more than 24 h or resulting in death [1].

Despite improvements in the treatment of acute stroke, strokes continue to represent the third-leading cause of death in many countries. Therefore, identification and prevention of stroke risk factors are gaining increasing importance. Approximately 70% of stroke risk is attributed to known etiological risk factors, and genetic factors may account for a significant proportion among the remaining unidentified causes [2].

 Polymorphisms affecting various genes, such as angiotensin converting enzyme (ACE), thrombin-activatable fibrinolysis inhibitor (TAFI), and nitric oxide synthase (NOS) genes, as suggested by recent advances in genetic research techniques are associated with the incidence of ischemic stroke (3–9). Nitric oxide (NO), an enzyme that regulates the endothelial microenvironment by reducing intracellular calcium levels, inhibits platelet aggregation and vascular smooth muscle cell proliferation, and reduces leukocyte adhesion [4,10].

The enzyme NOS catalyzes a reaction whereby L-arginine is synthesized from the N-terminus of guanidine. There are two forms of NOS: constitutive and inducible. Different genes synthesize different forms of NOS [NOS1 (neuronal NOS), NOS2, and NOS3 (endothelial NOS)] enzymes [11]. The NOS also inhibits the oxidation of low-density lipoprotein, thereby regulating the endothelial microenvironment [4,10]. Thus, any genetic problem disrupting the amount and structure of NO will cause an inclination towards clotting.

In some populations, although a significant relationship was found between the risk of ischemic stroke and the polymorphisms of the endothelial NOS (eNOS) gene (G894T, VNTR, T786C, etc.), studies to determine whether it may be a risk factor have delivered conflicting results. Studies in different populations with various polymorphisms may help determine the risk of stroke in those populations. We therefore aimed to determine whether there are significant differences in the G894T [rs1799983], intron 4 [27-bp TR] VNTR, and T786C [rs2070744] polymorphisms of the eNOS gene between stroke patients and healthy individuals in the central Anatolian population.

## 2. Materials and methods

### 2.1. Identification of cases, inclusion and exclusion criteria

To investigate whether there are significant differences in the distributional frequencies of the G894T (rs1799983), intron 4 (27-bp TR) VNTR, and T786C (rs2070744) polymorphisms of eNOS in the central Anatolian population, we designed two groups: healthy individuals (control group) and adult stroke patients aged > 18 years. We included 272 individuals (112 patients and 160 controls) in the study. Individuals without a history of ischemic stroke were included in the control group. Patients with ischemic stroke were classified according to the Trial of Org 10172 in acute stroke treatment (TOAST) classification system, and those with “stroke of undetermined etiology” and without any of the exclusion criteria were included in the patient group. The exclusion criteria are listed in Figure. We collected blood samples from the control and the patient groups upon their admission to the neurology outpatient clinic or emergency department of our hospital.

**Figure:**
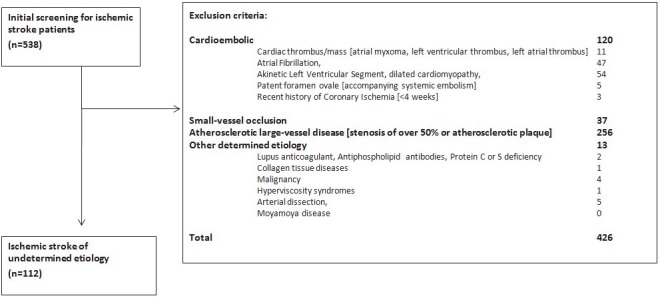
The flow chart of the patients.

We recorded demographic information including age, sex, medical history, and risk factors for stroke such as hypertension, coronary artery disease, diabetes, hyperlipidemia (Table 1). Blood samples obtained from the patients and the control groups were stored at −80 °C and subsequently subjected to genetic analysis.

**Table 1:** Demographic data and the risk factors in patient and control groups.

		Patient [n, %]	Control [n, %]	P	χ²
Age [mean, standard deviation]		68.58 [± 10.848]	61.44 [± 8.104]	[< 0.05]	85.810
Gender	Male	62 55.4%	65 40.6%	< 0.05	6.779
	Female	50 44.6%	95 59.4%	< 0.05	6.779
Diabetes mellitus		40 35.7%	33 20.6%	< 0.05	8.011
Hypertension		61 54.5%	47 29.4%	< 0.05	15.785
Coronary artery disease		15 13.4%	47 29.4%	< 0.05	11.321
Smoke		9 8%	15 9.4%	NS	1.159
Hyperlipidemia		32 28.6%	19 11.9%	NS	2.449

NS: Nonsignificant

### 2.2. DNA isolation

We isolated genomic DNA from the blood samples using the DNA isolation kit (DNeasy Blood & Tissue Kit, Qiagen GmbH, Hilden, Germany). We checked the purity and quantity of the extracted DNA by measuring the absorbance at 260 nm and 280 nm using a spectrophotometer (Nano Drops spectrophotometer, Thermo Scientific, USA).

### 2.3. Real-time polymerase chain reaction (RT-PCR) stage

We performed real-time PCR using fluorescence-labeled TaqMan probes specific for the eNOS G894T [rs1799983], intron 4 (27-bp TR) VNTR, and T786C (rs2070744) polymorphisms. We mixed the hybridization solution (Taq polymerase, reaction buffer, and nucleotide mix), MgCl2, primers, and probes and genomic DNA so as to obtain a total volume of 20 μL and transferred them to a real-time PCR device. We also used positive and negative controls. After completion of the real-time PCR amplification, we generated melting curves and then genotyped eNOS G894T (rs1799983), intron 4 (27-bp TR) VNTR, and T786C (rs2070744) polymorphisms using an allelic discrimination assay.

### 2.4. Statistical analysis

We performed statistical analyses using the SPSS 15.0 (SPSS Inc., Chicago, IL, USA). We determined the mean and standard deviations of the continuous variables and the frequencies and percentages of the categorical variables. We used the chi-square and Fisher’s exact test for intergroup comparisons of categorical variables. We used binary logistic regression analysis to compare the frequencies of alleles among the patient and control groups. We used the wild type (of genotypes and alleles) as a reference for regression analysis.****P ≤ 0.05 was considered statistically significant.

## 3. Results

The mean age was 68.58 (± 10.848) years in the patient group and 61.44 (± 8.104) years in the control group. The patient group comprised 50 (44.6%) females and 62 (55.4%) males, whereas the control group comprised 95 (59.4%) females and 65 (40.6%) males. The demographic properties and risk factors of the patients have been presented in Table 1.

### 3.1. G894T polymorphism

Table 2 presents the genotypes and allele frequencies. The differences in both genotypes and T alleles were not statistically significant between the patient and control groups (Table 2).

**Table 2:** Genotype distributions of eNOS G894T [rs1799983], intron 4 [27-bpTR] VNTR, eNOS T786C polymorphisms, and allelic frequencies in the patient and control groups [binary logistic analysis, wild type is reference for statistical analysis].

Polymorphism			Patient [n, %]	Control [n, %]	P	OR	95% CI
G894T [rs1799983]	Genotype	GG	21 [%18.8]	38 [%23.8]	-	1	
		GT	77 [%68.8]	103 [%64.4]	0.262	1.440	(0.76-2.77)
		TT	14 [%12.5]	19 [%11.9]	0.561	1.313	(0.52-3.29)
	Allele	G	115 [%39.1]	179 [% 60.9]	-	1	
		T	109 [%43.8]	140 [%56.2]	0.272	1.211	(0.86-1.70)
intron4 VNTR	Genotype	4b/b	57 [%50.9]	124 [%77.5]	-	1	
		4b/a	50 [%44.6]	34 [% 21.3]	< 0.0001*	3.396	(1.93-6.08)
		4a/a	5 [%4.5]	2 [%1.3]	0.016*	10.631	(1.51-215.9)
	Allele	b	164 [%36.8]	282 [% 63.2]	-	1	
		a	60 [%61.2]	38 [%38.7]	< 0.0001*	2.715	(1.73-4.26)
T786C	Genotype	TT	59 [%52.7]	38 [%23.8]	-	1	
		TC	35 [%31.2]	103 [% 64.4]	< 0.0001**	0.244	(0.13-0.44)
		CC	18 [%16,1]	19 [%11.9]	0.314	0.663	(0.30-1.48)
	Allele	T	152 [%45.92]	179 [%54.07]			
		C	72 [%33.96]	140 [%66.03]	0.006**	0.605	(0.42-0.86)

*The frequency of 4b/a, 4a/a genotypes and a allele were significantly higher in the patient group than the control group. **The frequencies of TC genotype and C allele were significantly lower rate in the patient group than the control group.

### 3.2. Intron 4 (27-bpTR) VNTR (4b/a) polymorphism

The 4b/a (heterozygous polymorphism) and 4a/a (homozygous polymorphism) genotypes as well as the a alleles were significantly higher in the patient group than in the control group (OR: 2.715, 95% Cl: 1.73–4.26, P < 0.001; OR: 3.396, 95% Cl: 1.93–6.08, P < 0.001; OR: 10.631, 95% Cl: 1.51–215.89, P = 0.016, respectively) (Table 2).

### 3.3. T-786C polymorphism

Table 3 also presented the results of the binary logistic regression analysis. Unlike the first two polymorphisms, the wild type genotype and allele (TT and T) were found to be higher in the patient group than in the control group. The TC genotype and C alleles of the T786C (rs2070744) polymorphism was significantly less frequent in the patient group than in the control group (OR: 0.244, 95% Cl: 0.13–0.44, P < 0.001; OR: 0.605, 95% Cl: 0.42–0.86, P = 0.006, respectively) (Table 2).

**Table 3:** Statistical results of studies conducted on enos t786c [rs2070744] polymorphism.

	Genotype			Allele		Country (Ethnicity)
	TT	TC	CC	T	C	
Yao YS et al.	NS	NS	NS	NS	NS	Asian, Caucasian
Guo X	NS	NS	NS	NS	NS	Asian, Caucasian
Wang M et al.	NS	P < 0.05 *	NS	NS	NS	Asian
Our study	NS	< 0.0001*	NS	NS	0.006*	Anatolian

NS: Statistically nonsignificant result. TT, TC, CC: Genotypes of eNOS T786C polymorphism. T and C: Alleles of eNOS T786C polymorphism. * The frequency of TC genotype and C allele were significantly lower rate in the patient group than the control group (protective effect?).

## 4. Discussion

Some studies on eNOS gene polymorphisms (including G894T, VNTR, and T786C) have suggested that, in some populations, eNOS is a risk factor for ischemic stroke; however, few conflicting results have also been reported [3,4,6,9,10,12–16]. Polymorphisms may play a specific penetrating role for ethnicity in the sensitivity of ischemic stroke. We investigated the existence of a relationship between the G894T (rs1799983), VNTR (4b/a), and T786C (rs2070744) polymorphisms of the eNOS gene and ischemic stroke in the central Anatolian population.

### 4.1. T-786C polymorphism

According to some metaanalyses, no significant differences exist between the Asian and Caucasian populations in terms of the T786C genotypes and allele distributions (Table 3) [6,10]. However, in a contrasting metaanalysis reported by Wang et al. [17], the predominance of the C allele of the T-786C polymorphism was highlighted in Asians, indicating a significant protective effect against the risk of ischemic stroke in the codominant models (C vs T for T-786C) (Table 3) [17]. In our study, we also observed that the C alleles and TC heterozygote polymorphism genotype were significantly lower in the patient group (with the possibility of a protective effect) in the Anatolian population (Tables 2 and 3).

### 4.2. G894T polymorphism

A significant relationship between the T allele and TT genotype of the G894T polymorphism and ischemic stroke risk was found in Asians and north Indians, but not in Caucasians (Table 4) (3,6,10,17,18). In Morocco, Diakite et al. reported a significant relationship between the G894T eNOS polymorphism and ischemic stroke in recessive, dominant, and codominant models (Table 5) [16].

**Table 4:** Statistical results of studies conducted on enos g894t polymorphism.

	Genotype			Allele		Country (Ethnicity)
	GG	GT	TT	G	T	
Yao YS et al.	NS	NS	NS	NS	Asians P < 0.05*Caucasians NS	Asian, Caucasian
Diakite B et al.	NS	P < 0.05*	P < 0.05*	NS	NS	North African
Hong-miao T et al.	NS	NS	P < 0.05*	NS	NS	White, East Asian
Guo	NS	NS	P < 0.05*	NS	Asians P < 0.05*Caucasians NS	Asian, Caucasian
Kumar A et al.	NS	NS	P < 0.05*	NS	NS	North Indian
Wang M et al.	NS	NS	NS	NS	Asian P < 0.05*	Asian, Caucasian
Guldiken B et al.	NS	NS	NS	NS	NS	Anatolian
Our study	NS	NS	NS	NS	NS	Anatolian

NS: Statistically nonsignificant result. GG, GT, TT: Genotypes of eNOS G894T Polymorphism. G and T: Alleles of eNOS G894T polymorphism. *Patients have significantly higher rate than controls.

**Table 5:** Statistical results of studies conducted on enos intron 4 vntr polymorphism.

	Genotype			Allele		Country (Ethnicity)
	4b/b	4b/a	4a/a	4b	4a	
Munshia A	NS	NS	P < 0.05*	NS	P < 0.05*	Indian
Yao YS et al.	NS	NS	NS	NS	P < 0.05*	Asian, Caucasian
Wang M et al.	NS	NS	NS	NS	Asians P < 0.05* Caucasians NS	Asian, Caucasian
Guo X	NS	NS	Asians P < 0.05*Caucasians NS	NS	P < 0.05*	Asian, Caucasian
Yemişçi M.	NS	NS	P < 0.05**	NS	NS	Anatolian
Our study	NS	P < 0.0001*	P: 0.047*	NS	P < 0.0001*	Anatolian

NS: Statistically nonsignificant result. 4b/b, 4b/a, 4a/a: Genotypes of eNOS 4 VTNR polymorphism. 4a and 4b: Alleles of eNOS 4 VTNR polymorphism. *Patients have significantly higher rate than controls. **Patients have significantly lower rate than controls (protective?).

In a study conducted by Guldiken et al. [19], the difference in the allelic frequency and genotypic distribution of the G894T (rs1799983) polymorphism between the ischemic stroke and the control group in the Anatolian population was not statistically significant (Table 4) [19]. This result was similar to our results in the Anatolian population (Table 4).

### 4.3. Intron 4 (27-bpTR) VNTR (4b/a)

Some studies have reported a significant relationship between ischemic stroke and the 4a/a genotype and/or the 4a allele of intron 4 (27-bpTR) VNTR polymorphism of the eNOS gene in South India and among the Asian population, but not in the Caucasian population (Table 5) [4,6,10,17].

Wang et al. reported a significant relationship between the 4a allele and the risk of ischemic stroke in dominant and codominant models in the Chinese population (Table 5) [17].

Yemişçi et al. [20] suggested that, in the Anatolian population, the 4a/a genotype is protective for isolated symptomatic lacunar infarction, and this effect is enhanced in the absence of the T786C polymorphism in the promoter region. On the contrary, in our study, the frequency of the 4 a/a and 4b/a genotypes and alleles were higher in the patient group than in the control group, and this difference was highly statistically significant. This contrasting result may be attributed to the inclusion of only the lacunar infarction group by Yemişçi et al. (Table 5) [20].

Our study is limited by the absence of the evaluation of body mass index and cholesterol subtypes of patients. According to the TOAST classification, the incidence of stroke with an unknown cause is 25%–39% [20]. We selected only a specific subset of patients and excluded other causes of stroke, such as atrial fibrillation and we couldn’t add some important polymorphisms such as factor V and prothrombin gene polymorphisms for analysis, because they are not routinely available in our laboratory.

In conclusion, this is the first study on the eNOS T786C polymorphism and the second study on the G894T [rs1799983] and intron 4 VTNR [4a/a] gene polymorphisms in the Anatolian population. While no significant relationship exists between the T786C polymorphism and stroke in Asians and Caucasians, we found a significantly low frequency of the T786C polymorphism (which has a protective effect) in Anatolian stroke patients, which is similar to that reported by Wang et al [17]. Guldiken et al. [19], too, found no relationship between the eNOS G894T [rs1799983] gene polymorphism and stroke risk in the Anatolian population compared with the Asian population. We also detected a highly significant increase in the intron 4 VTNR gene polymorphism in the stroke patients compared with the Caucasian population. On this basis, further studies of the normal function of NO are warranted in terms of primary and secondary protection against ischemic stroke.

## Acknowledgements

The study was approved by the local ethic committee of Meram Medical Faculty, Ethic approval code: 2015/396. All participants provided informed consent in the format required by the relevant authorities and/or boards.
